# Etymologia: *Ehrlichia*

**DOI:** 10.3201/eid1812.ET1812

**Published:** 2012-12

**Keywords:** etymologia, Ehrlichia, bacteria, Paul Ehrlich

## Ehrlichia [ār-lik′e-ə]

Named in honor of German scientist Paul Ehrlich, *Ehrlichia* is a genus of gram-negative bacteria of the family *Anaplasmataceae*. Although Ehrlich was not a bacteriologist and was primarily known for his work in hematology, immunology, and chemotherapy, the species *Ehrlichia kurlovi* was proposed in 1937 by Russian rickettsiologist Sh. D. Moshkovsky.

In 1889, Mikhail Georgiyevich Kurloff, perhaps working with Ehrlich, published a description of atypical granules in guinea pig leukocytes. These granules were assumed to be normal; rickettsiae would not be discovered for several decades. In his 1937 paper, Moshkovsky recognized the guinea pig granules as rickettsial inclusion bodies and named the genus “in honor of Paul Ehrlich, since it was in his laboratory that the first representatives of this group were discovered, and because he has contributed so much to the study of the morphology of the blood and of the agents of infectious diseases.”
